# Upregulated Genes In Sporadic, Idiopathic Pulmonary Arterial Hypertension

**DOI:** 10.1186/1465-9921-7-1

**Published:** 2006-01-03

**Authors:** Alasdair J Edgar, Matilde R Chacón, Anne E Bishop, Magdi H Yacoub, Julia M Polak

**Affiliations:** 1 Department of Craniofacial Development, King's College, London, SE1 9RT, UK; 2 Hospital Universitari de Tarragona Joan XXIII, Unitat de Recerca, C/Dr. Mallafre Guash, 4, 43007 Tarragona, Spain; 3 Tissue Engineering and Regenerative Medicine Centre, Faculty of Medicine, Imperial College, London SW10 9NH, UK; 4 Heart Science Centre, Imperial College, Harefield, Middlesex, UB9 6JH, UK

## Abstract

**Background:**

To elucidate further the pathogenesis of sporadic, idiopathic pulmonary arterial hypertension (IPAH) and identify potential therapeutic avenues, differential gene expression in IPAH was examined by suppression subtractive hybridisation (SSH).

**Methods:**

Peripheral lung samples were obtained immediately after removal from patients undergoing lung transplant for IPAH without familial disease, and control tissues consisted of similarly sampled pieces of donor lungs not utilised during transplantation. Pools of lung mRNA from IPAH cases containing plexiform lesions and normal donor lungs were used to generate the tester and driver cDNA libraries, respectively. A subtracted IPAH cDNA library was made by SSH. Clones isolated from this subtracted library were examined for up regulated expression in IPAH using dot blot arrays of positive colony PCR products using both pooled cDNA libraries as probes. Clones verified as being upregulated were sequenced. For two genes the increase in expression was verified by northern blotting and data analysed using Student's unpaired two-tailed *t*-test.

**Results:**

We present preliminary findings concerning candidate genes upregulated in IPAH. Twenty-seven upregulated genes were identified out of 192 clones examined. Upregulation in individual cases of IPAH was shown by northern blot for tissue inhibitor of metalloproteinase-3 and decorin (*P *< 0.01) compared with the housekeeping gene glyceraldehydes-3-phosphate dehydrogenase.

**Conclusion:**

Four of the up regulated genes, magic roundabout, hevin, thrombomodulin and sucrose non-fermenting protein-related kinase-1 are expressed specifically by endothelial cells and one, muscleblind-1, by muscle cells, suggesting that they may be associated with plexiform lesions and hypertrophic arterial wall remodelling, respectively.

## Background

In pulmonary hypertension (PAH), the mean resting pulmonary arterial pressure is greater than 25 mm Hg in contrast to the normal adult pulmonary circulation which has little resting vascular tone. The disease is also characterised by an absence of a significant pulmonary vasodilator response [1]. Many precapillary pulmonary arteries are affected by plexiform lesions, medial hypertrophy, intimal fibrosis and microthrombosis. PAH is a rare, often fatal condition, which progresses rapidly often leading to right-sided heart failure if untreated. It has a prevalence of less than two in a million and is more common in females. Most cases are idiopathic (IPAH), but about 6% are hereditary, with the major familial PAH (FPAH) locus located at 2q31. This is an autosomal dominant disease with incomplete penetrance. About 50% of these PAH families have been shown to have mutations in the bone morphogenetic protein receptor-II gene (BMPR2), but only 10% of cases of the sporadic or idiopathic form of PAH are associated with germline mutations in BMPR2 [2]. Additionally, mutations in activin-like kinase type-1 (ALK-1), another transforming growth factor-beta (TGF-β) receptor family member, have also been found in some patients with hereditary haemorrhagic telangiectasia and PAH [3]. A microarray study of differential gene expression in PAH has shown that there are distinct distinguishing patterns between IPAH and FPAH [4].

The hypothesis for this study was that the identification of differential gene expression in IPAH patients who were not known to have mutations in BMPR2 may help to elucidate its pathogenesis and provide candidate target genes for therapeutic intervention. More specifically, we used tissue from cases of IPAH containing plexiform lesions [5] for this study to identify genes involved in the phenotypically abnormal endothelial cell proliferation found in this disease. To achieve this we used suppression subtraction hybridisation, a method by which rare differentially expressed transcripts can be enriched a thousand-fold [6] to generate a cDNA library enriched in genes upregulated in tissue taken from the peripheral lung of IPAH patients.

## Methods

### Patients and tissues

Peripheral lung samples were obtained immediately after removal from patients undergoing lung transplant for IPAH at Harefield Hospital, Middlesex, U.K. and control tissues consisted of similarly sampled pieces of donor lungs not utilised during transplantation, as previously described [7] with the approval of the ethics committee of Hillingdon Area Health Authority. The control lung donors had no systemic disease and were free of known infections before surgery and liver and renal diseases were specifically excluded by biochemical analyses. The mean age of the IPAH patients (n = 4; 2 M, 2 F) was 43 years (range between 28 and 52 years) and that of the control donors (n = 4; 2 M, 2 F) was 40 years (range between 18 and 57 years). No evidence of BMPR2 mutations were found in the IPAH patients used in this study [8]. Tissues were snap frozen in liquid nitrogen and stored at -70°C, for subsequent RNA extraction. Frozen sections from adjacent tissue blocks fixed in 4% paraformaldehyde were stained with haematoxylin and eosin or immunostained.

### Isolation of RNA from tissues, cells and cDNA synthesis

For SSH total RNA was isolated, from approximately 1.0 g frozen tissues using the RNeasy method (Qiagen Ltd., Crawley, UK). The concentration and purity of eluted RNA was determined spectrophotometrically (O.D. 260/280 ratio between 1.8–2.0) and the quality of the RNA verified by denaturing agarose gel electrophoresis (28 S/18 S ratio between 1.5–2.5). Equal amounts of total RNA from 4 cases of PPH and 4 control lung tissues (80 μg each) were pooled. From the pooled total RNA, poly(A)^+ ^RNA was isolated using the PolyATtract mRNA purification procedure (Promega). Double stranded cDNA was synthesised from poly(A)^+ ^RNA, using the PCR-select cDNA subtraction kit as described in the manufacturer's instructions (Clontech). For the analysis of TIMP-3 expression in cultured human cell isolates and cell lines, total RNA was extracted using guanidine thiocyanate and treated with DNase-I to remove any contaminating genomic DNA (SV total RNA isolation system, Promega Southampton, U.K.). Total RNA was reverse transcribed with an oligo-dT primer using an AMV RNase H- reverse transcriptase (ThermoScript, Life Technologies, Paisley, U.K.). The human TIMP-3 primers were designed to amplify the 633 base pair ORF. The primers were; sense 5'-ATGACCCCTTGGCTCGGGCTCAT-3' (exon 1) and antisense 5'-GGGGTCTGTGGCATTGATGATGCTT-3' located on exons 1 and 5 respectively. The human glyceraldehyde-3-phosphate dehydrogenase (G3PDH) primers were; sense 5'-CATCACCATCTTCCAGGAGC-3' (exon 4) and antisense 5'-ATGCCAGTGAGCTTCCCGT-3' (exon 8) which gave a 474 base pair product. As a negative control reverse transcriptase was omitted from the RT reaction. PCR was carried out on a thermocycler (PE Applied Biosystems 2400) using Taq Gold polymerase (PE Applied Biosystems, Warrington, U.K.) and cDNA from 175 ng of total RNA. Amplification was for 32 cycles for TIMP-3 and 27 cycles for G3PDH. PCR products were examined by agarose gel electrophoresis and stained with ethidium bromide.

### Generation of a IPAH subtracted library by SSH

SSH was performed using the PCR-Select Subtraction protocol (Clontech) according to the manufacturer's recommendations. Briefly, double stranded cDNA from the primary pulmonary hypertensive pool was used as the tester and cDNA from the donor pool used as the driver using a ratio of 1:30. Differentially expressed genes were amplified from the subtracted cDNA using suppression PCR. A GeneAmp 2400 (PE Biosystems) was used for thermal cycling. The conditions used were 94°C for 10 sec, 68°C for 30 sec and 72°C for 90 sec, for the initial 30 cycle PCR and a further 12 cycles for the nested PCR. The level of expression of the housekeeping gene glyceraldehydes-3-phosphate dehydrogenase (G3PDH) in the subtracted library was used to determine the efficiency of subtraction using an RT-PCR assay. The expression of G3PDH in the subtracted library was only detected after 33 cycles of PCR, whereas in the unsubtracted library it was detected after 18 cycles indicating that the library was subtracted efficiently. The IPAH subtracted library was size fractionated on SizeSep 400 spun columns (Amersham Pharmacia Biotech) to select for longer, more informative, cDNAs greater than 400 basepairs.

### Cloning and colony PCR

The IPAH subtracted cDNA library was ligated into the pCR-II-TOPO vector (Invitrogen), a TA cloning system, and transformed into One Shot TOP10 competent cells (Invitrogen). The subtracted library was plated on to LB medium agar plates supplemented with 50 μg/mL ampicillin and treated with 40 mg/mL 5-bromo-4-chloro-3-indol-β-D-galactopyranoside (Promega) dissolved in dimethylformamide. Agar plates were incubated overnight at 37°C. Positive colonies containing inserts were inoculated in 100 μL LB medium containing ampicillin. Plasmids containing cloned sequences from the subtracted library were identified using a colony PCR protocol based on the PCR-Select Differential Screening protocol (Clontech). Briefly, cloned inserts were amplified by PCR and the products analysed by agarose gel electrophoresis.

### Verification of differential IPAH gene expression using dot blot arrays (reverse Northern blot) of positive colony PCR products

Each positive colony PCR product was denatured in an equal volume of 600 mM NaOH and 0.5% bromophenol blue and 2 μL dot blotted on to a Hybond-N nylon membrane (Amersham Pharmacia Biotech). β-actin and G3PDH control cDNAs were also applied. Membranes were prepared in duplicate, neutralised in 500 mM Tris pH 7.4 for 5 min, washed with water and UV cross linked. Dot blot membranes were pre-hybridised in express hybridisation solution (Clontech) supplemented with 1% blocking solution (100 μg/mL denatured sheared salmon sperm DNA in 0.2 × SSC) for 1 hour at 72°C. The membranes were hybridised with random primed ^32^P-dCTP labelled probes from either the subtracted or unsubtracted library as described in the PCR-select differential screening protocol (Clontech) and hybridised overnight at 72°C. Membranes were washed 4 times with 2 × SSC, 0.5% SDS at 68°C for 20 min followed by 2 stringency washes with 0.2 × SSC, 0.5% SDS at 68°C for 20 min. Membranes were exposed to X-ray film with intensifying screens from 12 hours to 2 days at -70°C.

### Sequencing positive clones

Plasmid DNA was extracted from clones which were differentially expressed on the gene expression dot blot arrays, using the Wizard SV minipreps DNA purification system (Promega). Plasmids were sequenced using the Sp6 and T7 promoter primers (5'-ATTTAGGTGACACTATA-3' and 5'-TAATACGACTCACTATAGGG-3' respectively) with the big dye terminator cycle sequencing ready reaction kit containing AmpliTaq DNA polymerase, FS on an ABI 373XL Stretch Sequencer (PE Applied Biosystems). Sequences were compared with the GenBank database using a BLASTN search.

### RNA blot analysis

Total RNA was separated by denaturing formaldehyde gel electrophoresis, transferred to Hybond-N nylon membrane and hybridised with ^32^P-labelled probes of either an *Eco*RI insert of DNA from the SSH clones or a 0.8 kb *Eco*RI/*Hind *III insert of G3PDH. Membranes were pre-hybridised for 1 hour and hybridised overnight in a buffer containing 5 × SSC, 5 × Denhardt's solution, 0.5% SDS at 60°C. Post-hybridisation washes consisted of 2 × SSC for 10 min at room temperature, followed by 2 stringency washes with 0.2 × SSC and 1% SDS for 20 min at 65°C. Membranes were then exposed to film for 1–7 days at -70°C with intensifying screens and the resultant autoradiograms were quantified using Scion Image software (Scion Corporation, Maryland, U.S.A.).

### Immunocytochemistry

For immunocytochemistry, endogenous peroxidase was blocked with a solution of 0.03% (v/v) hydrogen peroxide in methanol for 20 min followed by washing (3 × 5 min) in phosphate-buffered saline (PBS). After incubation with normal goat serum diluted 1:30 for 30 min to block non-specific binding associated with the secondary antibody, sections were incubated overnight at 4°C with a rabbit antiserum raised to a synthetic carboxy-terminal peptide of human TIMP-3 diluted 1:500 (Chemicon International Inc. California, USA). Immunoreaction sites were visualised using an anti-rabbit biotinylated secondary antibody and the avidin-biotin-peroxidase complex procedure (Vector Labs, Peterborough, UK). Peroxidase activity was revealed with a solution of diaminobenzidine as chromogen with 0.2% (v/v) hydrogen peroxide in PBS to produce a brown reaction product and sections were counterstained with Harris' haematoxylin. Controls consisted of replacement of primary antibodies with non-immune rabbit serum. The sections were observed and photographed under a BH-60 microscope (Olympus, UK).

### Protein blotting

Protein was extracted from snap frozen peripheral lung samples in a ratio of 1 g to 4 mL of lysis buffer (50 mM Tris base, 2 mM EDTA and 50 mM NaCl, pH 7.4 with 1% (w/v) SDS) with added protease inhibitors (leupeptin 1 μg/mL, chymostatin 10 μg/mL, bestatin 40 μg/mL, pepstatin A 1 μg/mL, TLCK 50 μg/mL). Tissue was homogenised for 1 min using an Ultra-Turrax homogeniser (Janke & Kunkl, Staufen, Germany). Protein concentrations were determined using a microplate DC assay kit (BIO-RAD, Hemel Hempstead, UK). The absorbance was read at 750 nm against a bovine serum albumin standard curve. Extracted protein, standardised as 20 μg of total protein per sample, was electrophoresed through a 12% (w/v) SDS-polyacrylamide gel and transferred to a 0.45 μm nitro-cellulose membrane (Shleicher & Shuell, Dassel, Germany) for 2 h at 4°C, using a wet system (Bio-Rad, Hemel Hempstead, U.K.). Membranes were blocked overnight in 5% (w/v) non-fat dry milk, Tris-buffered saline with 0.1% Tween-20 (TBS-T). Membranes were incubated with primary rabbit antisera human TIMP-3 protein diluted 1:1000 in TBS-Tween for two hours at room temperature. This antisera shows no cross-reactivity with other TIMP family members. It recognises the glycosylated and unglycosylated forms of TIMP-3. Membranes were washed in TBS-T and then incubated for 1 hour with goat anti-rabbit antibody conjugated with horseradish peroxidase diluted 1:16,000. After repeated washes, the protein was detected using an enhanced chemiluminescence kit (ECL, Amersham International, Little Chalfont, U.K.) and films quantified using LabImage software (Kapelan Bio-imaging Solutions).

### Statistical analysis

Data were analysed using Student's unpaired two-tailed *t*-test. Statistical significance was accepted when *P *< 0.05.

## Results

All lung specimens from patients with IPAH showed characteristic vascular changes described as plexogenic pulmonary arteriopathy [9], including enlarged arteries with medial and/or intimal thickening, arteriolar muscularization, intimal fibrosis, dilatation and plexiform lesions. Control tissues showed no evidence of vascular or any other pathology. RNA was extracted and pooled from the tissues and SSH was performed. A subtracted library enriched in transcripts upregulated in IPAH was cloned and colonies were isolated. PCR products from 192 of these colonies were generated and used to make dot blot arrays for gene expression analysis. Duplicate arrays were probed with the unsubtracted IPAH and donor cDNA libraries. Densitometric analysis of the exposed films identified those colonies that were upregulated. These cDNA clones were sequenced. All sequences match currently known human genes. However, the sequences for EGLN1, PDK4, GPR107, CCNL1 and VPS35 cDNAs were novel [GenBank: AF334711, AF334710, AF376725, AF180920 and AF191298 respectively]. In total, 27 separate genes were identified as being upregulated in IPAH (Table 1). Housekeeping genes commonly used for normalization in molecular biology such as β-actin, G3PDH and tubulin were absent indicating successful subtraction. Of the upregulated SSH clones 52% included some protein coding sequence and 48% were in the 3'UTR. This shows the utility of this method for identifying differentially expressed genes with partial protein sequence information. With regards to chromosomal localisation, there were no clusters of upregulated genes and none mapped to the known FPAH loci at 2q31 (PPH2), 2q33 (BMPR2) and 12q13 (ALK-1). Our approach to identifying upregulated genes in IPAH did not identify changes in BMP2 receptor expression since it is reduced in IPAH [10].

**Table 1:** Summary of upregulated genes in IPAH showing gene abbreviation, accession number, location of SSH cDNA clone sequence on gene transcript, encoded protein name and chromosomal localisation.

**Gene**	**Accession**	**Clone**	**Protein**	**Chromosome**
**Extracellular Proteins**				
AMOTL2	NM_016201	3'UTR	Angiomotin like-2	3q21-q22
SPARCL1	NM_004684	Coding & 3'UTR	Hevin	4q22.1
DCN	NM_001920	5'UTR & coding	Decorin isoform A	12q21
TIMP3	NM_000362	3'UTR	Tissue inhibitor of metalloproteinase-3	22q12.3
FLJ23191	AAQ89180	3'UTR	VLLH2748	4q27
**Membrane Proteins**				
ROBO4	NM_019055	Coding & 3'UTR	Magic roundabout	11q24.2
THBD	NM_000361	3'UTR	Thrombomodulin	20p12-cen
CD9	NM_001769	Coding & 3'UTR	Cluster of differentiation-9	12p13.3
TM4SF1	NM_014220	Coding & 3'UTR	L6 antigen	3q21-q25
GPR107	AF376725	3'UTR	G protein-coupled receptor 107	9q34.11
**Nuclear**				
EGLN1	NM_022051 and AF334711	3'UTR	Hypoxia-inducible factor prolyl hydroxylase 2	1q42.1
TACC1	NM_006283	3'UTR	Transforming acidic coiled coil-1	8p11
MBNL1	NM_021038	3'UTR	Muscleblind-1	3q25
CCNI	NM_006835	Coding	Cyclin I	4q21.1
CCNL1	AF180920	Coding	Cyclin L1	3q25.31
NPIP	NM_006985	Coding	Nuclear pore complex interacting protein	16p13-p11
**Cytoplasmic**				
YWHAZ	NM_003406	3'UTR	Tyrosine 3-monooxygenase/tryptophan 5-monooxygenase activation protein, zeta polypeptide	8q23.1
DAB2	NM_001343	3'UTR	Disabled homolog 2	5p13
COPA	NM_004371	Coding & 3'UTR	α-coatomer protein	1q23-q25
VPS35	AF191298	Coding	Vesicle protein sorting 35	16q12
C14orf153	NM_032374	Coding	Chromosome 14 open reading frame 153	14q32.32-q32.33
**Enzymes**				
FMO2	NM_001460 and 3'UTR AK098145	3'UTR	Pulmonary flavin-containing monooxygenase 2/Dimethylaniline monooxygenase	1q23-q25
SNRK	NM_017719	3'UTR	Sucrose non-fermenting protein-related kinase-1	3p22.1
ASAH1	NM_177924 and NM_004315	Coding & 3'UTR	N-acylsphingosine amidohydrolase/acid ceramidase	8p22-p21.3
PDK4	NM_002612 and AF334710	3'UTR	Pyruvate dehydrogenase kinase-4	7q21.3-q22.1
ALDH1A1	NM_000689	Coding	Aldehyde dehydrogenase 1A1	9q21.13
MAOA	NM_000240	Coding & 3'UTR	Monoamine oxidase A	Xp11.23

**Figure 1:**
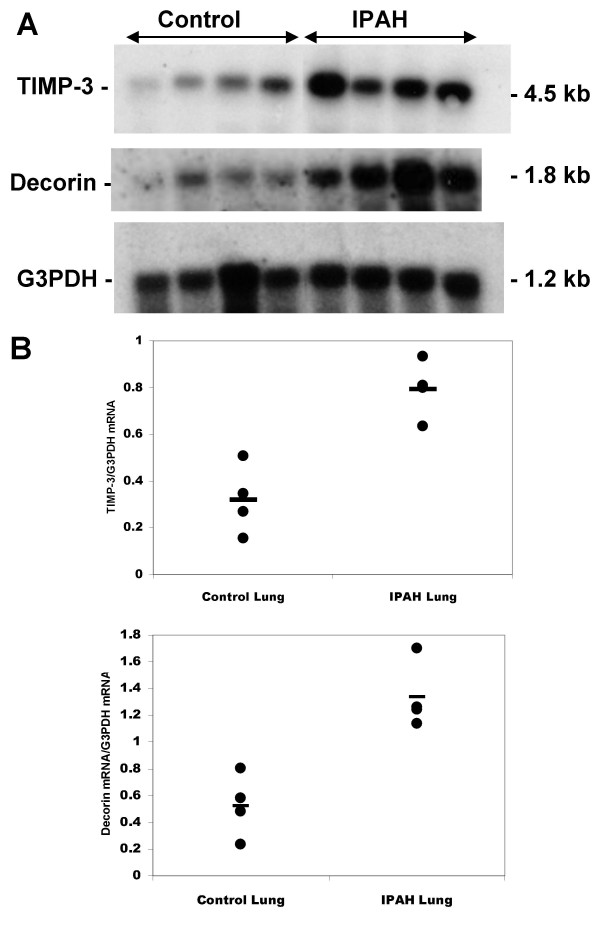
TIMP-3 and decorin mRNA expression in donor and IPAH lung. A total RNA blot of lung tissue from individual patients and donors was probed with the TIMP-3 and decorin clones isolated from the SSH library. G3PDH was used as a control housekeeping gene (A). Ratios of gene expression normalised relative to expression of G3PDH. Bars indicate mean expression values (B).

**Figure 2:**
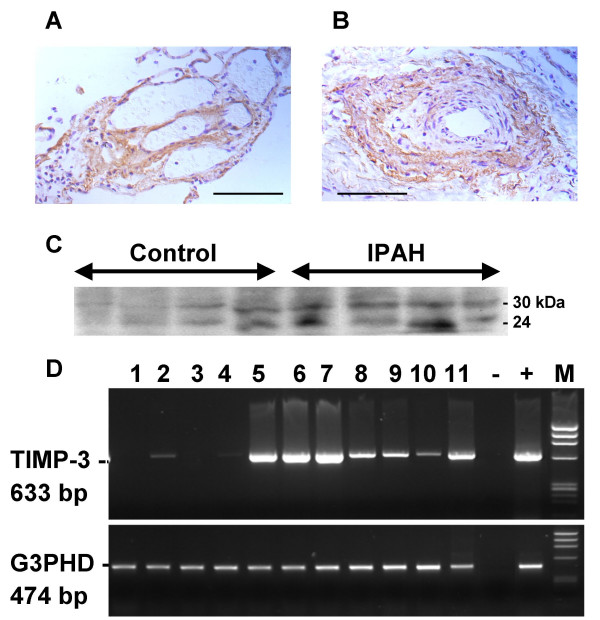
Localisation of TIMP-3 in IPAH lung tissue. Immunocytochemistry for TIMP-3 was carried out using an polyclonal antisera raised against the human carboxy-terminal peptide of TIMP-3. Plexiform lesion showing location of TIMP-3 in subendothelium. (A). Hypertrophied artery, showing location of TIMP-3 in in vascular smooth muscle cells and myoblasts (B). Scale bars = 100 μm. Protein extracted from IPAH and donor lung tissues was analysed by western blot using an antiserum to TIMP-3 (C). The antibody recognises both the unglycosalated form (24 kDa), and the glycosalated form of TIMP-3 (30 kDa). Expression of TIMP-3 in human cells (D). RNA extracted from human cell lines and explant derived cells was reverse transcribed and amplified by PCR and run on agarose/ethidium bromide stained gels. RT-PCR for TIMP-3 (top panel) and G3PDH (bottom panel). The cell types examined were: Lanes; 1, HL60 (promyelocytic leukaemia); 2, Daudi (Burkitt's lymphoma); 3, EB-transformed lymphocytes; 4, K562 (erythroleukemia) 5, pulmonary adult fibroblasts; 6, pulmonary artery smooth muscle; 7, bronchial smooth muscle; 8, A549 (adenocarcinoma alveolar epithelial); 9, H322 (adenocarcinoma bronchial epithelial); 10, placental microvascular endothelial; 11, umbilical vein endothelial; -, no cDNA negative control; + control lung cDNA; M, phiX174 DNA/HaeIII markers.

Since the upregulated genes were identified from pooled patient samples we verified increased expression of TIMP-3 and DCN in individual IPAH patient samples by RNA blot for two genes (Fig. 1). We observed an average 2.49 fold increase in TIMP-3 expression relative to the housekeeping G3PDH mRNA and a 2.55 fold increase in decorin expression (both *P *< 0.01). Expression relative to 28 S ribosomal RNA was found to be similarly increased (data not shown). Hypertrophied arteries displaying intimal proliferation from IPAH lung showed TIMP-3 staining in vascular smooth muscle cells and/or myofibroblasts (Fig. 2A). In plexiform lesions, TIMP-3 staining appeared to be located in the subendothelium (Fig. 2B). Lung sections incubated without the primary specific antibody were negative. TIMP-3 protein levels, determined by protein blotting, were increased, on average, 2.3 fold in IPAH relative to normal lung tissue for both the 24 kDa unglycosylated and the 30 kDa glycosylated forms of TIMP-3 (significantly for the 30 kDa isoform, *P *< 0.05; but not significantly for the 24 kDa isoform *P *< 0.08) (Fig 2C). To identify the cell types likely to be responsible for the expression TIMP-3 in lung tissue, expression levels in a number of human cultured cell lines were examined by RT-PCR (Fig. 2D). Both bronchial and pulmonary artery smooth muscle cells and adult lung fibroblasts expressed the highest levels of TIMP-3 and are likely to account for the majority of TIMP-3 expression in the lung. Two lung epithelial cell lines also expressed TIMP-3, but to a lesser extent. Two endothelial cell types, from placental microvessels and umbilical vein, also expressed TIMP-3. Non-adherent blood-derived cell lines showed little TIMP-3 expression. Taken together the northern and protein blotting data supports the validity of the experimental design and suggests that the genes identified by SSH and subsequent dot blot arrays are upregulated in IPAH.

## Discussion

Idiopathic pulmonary arterial hypertension (IPAH) is a pulmonary vasculopathy of unknown aetiology. While genetic studies have provided considerable progress in our understanding of IPAH through the identified mutations in the gene for BMP-RII in some patients with familial and sporadic IPAH, our study sought to elucidate changes in gene expression in lung tissues from patients with IPAH without known BMP-RII mutations. We used the PCR-based SSH method to identify upregulated genes in IPAH which may contribute to the disease process, lead to the discovery of the cause(s) of the disease and provide potential therapeutic targets for treatment intervention. With the aim of identifying genes associated with the arteriopathy we used lung tissue samples containing plexiform lesions in this study. Endothelial cells normally form a monolayer, but in plexiform lesions they display some properties of tumours, forming complex structures and are mostly monoclonal in origin in IPAH, but are mostly polyclonal in secondary PAH [11,12].

### Extracellular proteins

We found five genes for extracellular proteins upregulated in IPAH. Hevin (SPARCL1) was initially described as an acidic protein secreted by high endothelial venules inhibiting endothelial cell adhesion and focal adhesions forming and in so doing modulates adhesion to the basement membrane [13]. Hevin binds collagen I [14] and the other functions of hevin have been recently reviewed [15]. The upregulation of hevin expression in IPAH may be due to the abnormal vascular proliferation in the plexiform lesions where its anti-adhesive properties may be responsible for the loss of an endothelial monolayer resulting in a more rounded endothelial phenotype. The location of the single hevin gene is on chromosome 7, not on chromosome 4 as previously reported [16]. Hevin production is induced in response to focal mechanical injury [17], and in IPAH its increased expression may be due to the high pulmonary pressure. It has been suggested that hevin has a pro-angiogenic role [18].

The decorin (DCN) clone corresponded to transcript variant A1 that encodes the full-length protein, isoform A. Decorin is a pericellular matrix proteoglycan that binds to type I and type VI collagen fibrils [19]. Transforming growth factor-β (TGF-β) is a major inducer of extracellular matrix synthesis and decreases the production of decorin [20]. Conversely, decorin binds to and inhibits active TGF-β producing an antifibrotic effect [21]. Increased expression of decorin may lead to a reduction in the TGF-β signalling pathway in a manner similar to the defective BMPR-2 signalling responsible for FPAH. Increases in decorin expression are associated with capillary endothelial cells in a model of angiogenesis and in inflamed arterial walls [22,23]. Decorin expressing tumours suppress neovascularization by inhibiting vascular endothelial growth factor (VEGF) mRNA expression [24] which leads us to suggest that decorin may be induced during plexiogenesis. However, an increase in decorin expression in smooth muscle may also contribute to IPAH since over expression of decorin in arterial smooth muscle cells promotes the contraction of type I collagen and enhance arterial calcification [25,26].

Tissue inhibitor of metalloproteinases-3 (TIMP-3) is one of four TIMP proteins that are natural inhibitors of matrix metalloproteinases (MMP), a group of peptidases that regulate the degradation, composition and turnover of basement membranes and extracellular matrix. TIMP-3 is the only one strongly bound to the extracellular matrix (ECM)[27] which may limit its activity to areas close to the site of synthesis. TIMP-3 plays an important role in lung development since the TIMP-3 null mouse develops a lung phenotype of spontaneous air space enlargement, similar to that of pulmonary emphysema [28] and has decreased bronchiole branching during morphogenesis [29]. It is not surprising to find that TIMP-3 is upregulated in IPAH as extensive vascular and smooth muscle remodelling is an active and on-going process in IPAH. This upregulation of TIMP-3 is likely to alter the proteolytic balance between TIMP-3 and MMPs and, in so doing, is likely to contribute to vascular and smooth muscle remodelling in IPAH. Recently, a MMP-3/TIMP-1 imbalance was found in the smooth muscle cells obtained from hypertrophic IPAH arteries [30].

The function of angiomotin-like-2 (AMOTL2) is currently unknown. However, angiomotin (AMOTL1) binds the angiogenesis inhibitor angiostatin and loss of its COOH-terminal PDZ binding domain prevented the growth of haemangioendothelioma by angiostatin [31]. This indicates that AMOTL2 may play a similar role in regulating endothelial proliferation.

The VLLH2748 protein is a 568-residue protein of unknown function. A bioinformatic analysis suggests that it has an amino-terminal secretion signal with a likely cleavage site, but no transmembrane domain suggesting that it is a soluble extracellular protein. VLLH2748 has homology to the interleukin-6 signal transducer isoform 1 precursor (IL6ST) in the cytokine-binding homology region. This suggests that VLLH2748 may function to sequester an interleukin-like cytokine in the extracellular matrix and preventing it interacting with its receptor.

### Membrane proteins

We found five genes for membrane proteins upregulated in IPAH. Magic roundabout (ROBO4) is one of four roundabout type-1 transmembrane receptors in humans. Roundabout proteins 1,2 and 3 are neural guidance receptors that on binding Slit ligands mediate axonal repulsion. Their extracellular ligand-binding regions have five immunoglobulin cell-adhesion molecule domains and three fibronectin type III domains. ROBO4 differs by having two immunoglobulin domains followed by two fibronectin domains extracellularly and being specifically expressed on the surface of endothelial cells [32]. ROBO4 has been shown to bind Slit-2 [33,34], but another group did not find any interaction between ROBO4 and any of the three Slit ligands [35]. Given the sequence similarity between the Slit ligands and the Notch transmembrane receptors we suggest that Notch may also be a ligand for ROBO4. The Notch signalling network regulates interactions between physically adjacent cells and functions in regulating endothelial cell branching [36]. The Slit-2-ROBO4 interaction inhibits endothelial migration, partly through the extracellular-signal-regulated kinase (ERK) signalling pathway [34]. In adult mice ROBO4 is restricted to sites of active angiogenesis and is upregulated in response hypoxia [32,35]. ROBO4 over expression results in an increase in intersomitic blood vessel defects in zebrafish embryos [37]. Intriguingly, ROBO4 is upregulated in mice lacking the ALK-1 gene, which is mutated in some cases of FPAH, and these mice characteristically have aberrant fusion of endothelial tubes [33]. Overall the increased ROBO4 expression in IPAH lung may be associated with the presence of plexiform lesions. The apparently contradictory presence of ROBO4 at sites of active angiogenesis and its role in inhibiting endothelial migration remains to be resolved.

Thrombomodulin (TMBD) is an important inhibitor of blood coagulation. It is a type-1 membrane receptor that binds thrombin, resulting in the activation of protein C, which degrades clotting factors Va and VIIIa and reduces the amount of thrombin generated. Proteolytic cleave of TMBD from endothelial cells is thought to occur during vascular damage and soluble circulating levels of TMBD are elevated in hypertensive patients [38]. TMBD is regarded as being endothelial-specific, but treatment of smooth muscle cells by prostaglandins stimulates TMBD expression [39].

Two transmembrane 4 (tetraspanin) superfamily proteins were identified. Proteins of this superfamily are thought to interact laterally with each other forming microdomains on the cell surface. Cluster of differentiation-9 (CD9) modulates cell adhesion and lymphocyte transendothelial migration through interactions with fibronectin, and through its interaction with α6β1 integrin, regulates the formation of angiotubular structures [40]. CD9 also promotes muscle cell fusion and supports myotube maintenance [41]. The L6 antigen (TM4SF1) is highly expressed in lung carcinomas being involved in invasion and metastasis [42]. G-protein-coupled receptor 107 (GPCR107) is a seven transmembrane receptor of unknown function.

### Nuclear proteins

We found five genes that encode nuclear proteins upregulated in IPAH. The EGLN1 gene encodes the hypoxia-inducible factor prolyl hydroxylase 2 (PHD2), one of three closely related prolyl hydroxylases found in humans. PHD2 is the key oxygen sensor involved in setting a low steady-state level of HIF-1α in normoxia [43]. The hypoxia-inducible factor-1 (HIF-1) is a transcription complex that regulates many genes involved in the response to hypoxia, including vasodilation, and by upregulating VEGF-A induces angiogenesis. It is a heterodimer consisting of the short-lived HIF-1α and the constitutively expressed HIF-1β. PHD2 hydroxylates HIF-1α on key proline residues [44]. Hydroxylated HIF-1α subunits are recognised by and targeted for destruction in the proteasome by the von Hippel-Lindau tumour suppressor protein (VHL), an E3 ubiquitin ligase complex. Under hypoxia PHD2 activity is decreased and HIF-1α protein accumulates enabling the HIF-1 complex to activate transcription. The reason for the increase in expression of EGLN1 in IPAH is unknown. However, there is a feedback loop whereby the HIF-1 complex, formed under conditions of hypoxia, upregulates PHD2 [43]. Interestingly, the drug hydralazine is a vasodilator used in the treatment of severe hypertension and one of its mechanisms of action is to inhibit the enzymatic activity of PHD2 [45]. The PHD2 protein has two domains, a CT prolyl hydroxylase domain and an NT MYND zinc finger domain consisting of two fingers of the Cys_4_, CysHis_2_Cys type that is not present in the other two prolyl hydroxylases. The function of the MYND zinc finger domain of EGLN1 is not known, but the MYND domain of the myloid translocation gene on chromosome 8 (MTG8) binds to transcriptional corepressor proteins such as SMRT and histone deacetylases and does not act as a sequence specific DNA-binding transcription factor [reviewed in [46]]. We speculate that not only does PHD2 prevent transcription of hypoxia-induced genes by hydroxylating HIF-1α, but may also recruit corepressor proteins and histone deacetylases to hypoxia response elements (HRE).

The transforming acidic coiled-coil (TACC) proteins play a role in the spindle function, being localised to centrosomes where they interact with microtubules [47]. Fibroblasts transfected with TACC1 show cellular transformation and anchorage independent growth, [48], suggesting that inappropriate expression of TACC1 can impart a proliferative advantage.

Muscleblind (MBNL) proteins are required for the terminal differentiation of muscle and are recruited into ribonuclear foci, being depleted elsewhere in the nucleoplasm. MBNL1 is a nuclear zinc finger protein containing two pairs of zinc-fingers of the Cys_3_His type that may be involved in DNA binding. However, MBNL1 has an important role in RNA binding being recruited to the expansions of CUG repeats found in myotonic dystrophy in which the repeats form double-stranded RNA hairpins [49]. Interestingly, both cyclin L1 and MBNL1 are associated with regulation of mRNA splicing [50].

Cyclins function as regulators of cyclin-dependent kinases (CDK) and most show a characteristic periodicity in abundance through the cell cycle. Little is known about the functions of Cyclin I (CCNI) [51]. Cyclin L1 (CCNL1) is associated with the cyclin-dependent kinase 11p110 and is involved in regulating gene expression, RNA transcription and splicing [52,53].

The nuclear pore complex-interacting protein (NPIP) is a member of a large rapidly evolving family of primate specific proteins whose functions are unknown [54].

### Cytoplasmic proteins

Four genes encoding adaptor proteins with functions in intracellular transport were found to be upregulated in IPAH. They play various roles in a wide range of signal transduction pathways. Tyrosine 3-monooxygenase/tryptophan 5-monooxygenase activation protein, zeta polypeptide (YWHAZ) is an acidic adaptor and scaffolding protein that belongs to the 14-3-3 family of proteins. They mediate signal transduction by binding to phosphoserine-containing proteins changing their intracellular location [55]. YWHAZ plays a role in the control of cell adhesion and spreading by interacting with the platelet glycoprotein Ibα subunit of the glycoprotein Ib-V-IX complex that interacts with subendothelial von Willebrand factor to ensure recruitment of platelets at sites of vascular injury [56].

Disabled homolog 2 (Dab2) is a cystosolic cargo-specific adaptor protein, which binds to the cytoplasmic tails of lipoprotein receptors thereby selecting specific cargos at the plasma membrane during clathrin-coat assembly and lattice polymerization events [57]. It plays a role in a wide range of signal transduction pathways including the Wnt and eNOS pathways.

Transport of membrane proteins between the endoplasmic reticulum and Golgi compartments is mediated by COPI vesicles containing the coatomer protein-alpha (COPA)[58]. In addition to its role in intracellular transport COPA has a hormonal role. Proteolytic cleavage of the NT 25-residues of COPA forms xenin-25, a gastrointestinal hormone that stimulates exocrine pancreatic secretion. This peptide is related to neurotensin and both bind the neurotensin-1 receptor [reviewed in [59]]. In the lung, the neurotensin receptor is located mainly in fibroblasts [60]. Whether xenin-25 is produced by the lung and has a physiological role there remains to be determined.

Vesicle protein-sorting protein-35 (VPS35) is a core component of a large multimeric complex, termed the retromer complex, involved in recycling membrane receptor proteins from endosomes to the trans-Golgi network [61].

Chromosome 14 open reading frame 153 (C14orf153) encodes a 193-residue protein of unknown function that has a wide tissue distribution. The nematode homologue (accession number NP_741663) has been shown to bind to a gamma-glutamyltranspeptidase that plays important roles in the synthesis and degradation of glutathione and drug and xenobiotic detoxification [62].

### Enzymes

Sucrose non-fermenting protein (SNF1)-related kinase (SNRK) is a homologue of the yeast Snf1 kinase, mutants of which fail to thrive when provided with non-fermentable carbon sources. In the mouse lung SNRK expression is specific to capillary endothelial cells [63]. SNRK is phosphorylated and activated by the LKB1 serine/threonine-protein kinase that regulates cell polarity and functions also as a tumor suppressor [64]. In view of the vascular abnormalities found in LKB1 null mice LKB1 has been placed in the VEGF signalling pathway [65]. Together, these lines of evidence suggest that SNRK is also in the VEGF signalling pathway.

Monoamine oxidase A (MAOA) degrades amine neurotransmitters including serotonin. MAOA is located on the X chromosome and its upregulation in IPAH suggests a possible involvement in the development IPAH given the female prederiliction of the disease.

Acid ceramidase or N-acylsphingosine amidohydrolase (ASAH1) cleaves ceramide into sphingosine and a free fatty acid. Both ceramide and sphingosine are involved with lipid signal transduction. Ceramide is generally associated with growth arrest and apoptosis, whereas the sphingosine pathway is mitogenic [66].

Pyruvate dehydrogenase kinase 4 (PDK4) is one of four closely related PDKs found in humans. These isoenzymes regulate the activity of the pyruvate dehydrogenase complex (PDH) that catalyzes the oxidative decarboxylation of pyruvate and is located at the interface between glycolysis and the citric acid cycle. Phosphorylation of the E1alpha subunit of the PDH complex by a specific pyruvate dehydrogenase kinase (PDK) results in its inactivation. Pyruvate dehydrogenase kinase isozyme 4 is upreguated during hibernation where it inhibits carbohydrate oxidation resulting in anaerobic glycolysis using triglycerides as a source of fuel [67]. Starvation and diabetes also markedly increased the abundance of PDK4 mRNA especially in muscle tissues [68-70].

Aldehyde dehydrogenase 1A1 (ALDH1A1) is the cytosolic isoform of the second enzyme of the major oxidative pathway of alcohol metabolism. It may play a role also in retinoid synthesis in the bronchial epithelium and alveolar parenchyma, where it is upregulated by hypoxia [71,72].

Dimethylaniline monooxygenase 2 or pulmonary flavin-containing monooxygenase 2 (FMO2) is the major FMO enzyme in the lungs of many species, being localised to the pulmonary epithelium and plays a role in the in the oxidation of many xenobiotics and therapeutic drugs. About 25% of African-Americans have a at least one functional FMO2 allele [73], but in a majority of humans FMO2 encodes a non-functional truncated protein [74].

Overall, five genes, AMOTL2, VLLH2748, GPCR107, NPIP, C14orf153, that encode proteins of unknown function were identified and further studies using techniques such as in situ hybridization are required to elucidate their localization in the lung. These genes are candidates for further study in the pathology of IPAH and the formation of plexiform lesions.

Although mutations in the TGF-β family of receptors have been shown to play an important role in FPAH, DCN was the only gene found to be upregulated in IPAH that plays a role in the TGF-β signalling pathway.

A major cause for the elevated pulmonary vascular resistance in patients with IPAH is hypertrophic arterial wall remodelling caused by excessive pulmonary artery smooth muscle cell (PASMC) proliferation. MBNL was the only gene identified that is muscle specific and may play a role in the smooth muscle differentiation. However, SPARCL1, DCN and TIMP-3 may play significant roles in the matrix remodelling process that occurs in this disease. The upregulation of MAOA is of interest given the "serotonin" hypothesis of pulmonary hypertension. Serotonin has been shown to exert mitogenic effects on pulmonary artery smooth muscle cells and may contribute to the pulmonary vascular remodelling. Competitive inhibitors of the serotonin uptake transporters such as fluoxetine and paroxetine are used as appetite suppressants and their use increases the risk of IPAH [reviewed in [75]]. Indeed, the long allelic variant promoter of the serotonin transporters gene is associated with increased activity and confers susceptibility to IPAH [76]. Inappropriate smooth muscle and endothelial cell proliferation is a feature of IPAH and three genes, TACC1, CCNI and CCNL1, have been linked to cellular proliferation.

A previous study examining differential gene expression in PAH versus normal lung tissue using microarrays found that of 6800 genes assayed 2% were upregulated and 2.5% were downregulated [4]. Decorin was identified as being differentially expressed in both our SSH and this microarray study. Overall, the microarray expression patterns of IPAH samples were clearly distinct from those of normal lung tissues. The expression patterns of the two FPAH tissues more closely resembled those of normal tissues; however, they did not determine whether these FPAH patients had germline mutations in the BMPR2 gene. Because PAH may have an inflammatory component the expression patterns of peripheral blood mononuclear cells have also been examined by microarrays to identify possible markers of the disease [77]. Although discriminatory gene expression patterns were identified between PAH patients and normal individuals, no clear discriminatory patterns were identified between IPAH and secondary PAH diseases. Acid ceramidase was identified as upregulated in both our SSH and this microarray study, suggesting that upregulation of this gene may contribute to the inflammatory component of PAH (reviewed in [78]). In another microarray study of emphysema none of the genes found to be upregulated in IPAH by SSH were identified as being upregulated [79] emphasising the differences between the diseases. The extracellular protein hevin, which was upregulated in IPAH, was significantly down regulated in emphysema reflecting the tissue destruction present in this disease.

## Conclusion

We present preliminary novel findings concerning genes upregulated in IPAH which can be considered as candidate genes for further study in this disease. An aim of this study was to identify genes involved in plexiform lesions and our library was enriched in clones specifically associated with endothelial cells, a finding that gives support to the "disordered angiogenesis" hypothesis of IPAH [80]. Five genes, ROBO4, SNRK, TM4SF1, FMO2 and TIMP3, found in this screen have been preferentially associated with capillary endothelial cells from mouse lung [63] and TMBM expression was found specifically in tumour endothelial cells, but not normal endothelial cells [18]. Platelet thrombi are often found in plexiform lesions [5] and two genes, TM and YWHAZ, identified in this study are associated with blood coagulation. The VEGF glycoproteins are critical inducers of angiogenesis and would be expected to play a role in the formation of plexiform lesions. VEGF is expressed in plexiform lesions and its receptor VEGFR-2 and the HIF-1 transcription complex are overexpressed [80]. Although we did not identify these genes in this SSH screen, we found the key oxygen sensor, PHD2, which regulates the HIF-1 transcription complex, that subsequently regulates VEGF expression and we found a kinase, SNRK, that regulates a second kinase, LKB1, that is a component of the VEGF signalling pathway. Comparisons with other diseased control groups, such as secondary PAH with and without plexiform pulmonary arteriopathy would help clarify whether the pattern of differential gene expression found in this study is specific to IPAH and plexiform lesions. We cannot rule out the possibility that the upregulation of a number of these genes may be due to the pre-transplant medication of the patients. However, this study provides preliminary evidence for the involvement of candidate genes in IPAH.

## Competing interests

The author(s) declare that they have no competing interests.

## Authors' contributions

AJE, MRC, AEB and JMP participated in the design and coordination of the study; MHY obtained clinical material and clinical data; MRC carried out the SSH and northern blotting; AJE and MRC carried out the sequence analysis and AJE drafted the manuscript. All authors have read and approved the final manuscript.

## References

[B1] Wood P (1958). Pulmonary hypertension with special reference to the vasoconstrictive factor. Br Heart J.

[B2] Newman JH, Trembath RC, Morse JA, Grunig E, Loyd JE, Adnot S, Coccolo F, Ventura C, Phillips JA, Knowles JA, Janssen B, Eickelberg O, Eddahibi S, Herve P, Nichols WC, Elliott G (2004). Genetic basis of pulmonary arterial hypertension: current understanding and future directions. J Am Coll Cardiol.

[B3] Harrison RE, Flanagan JA, Sankelo M, Abdalla SA, Rowell J, Machado RD, Elliott CG, Robbins IM, Olschewski H, McLaughlin V, Gruenig E, Kermeen F, Halme M, Raisanen-Sokolowski A, Laitinen T, Morrell NW, Trembath RC (2003). Molecular and functional analysis identifies ALK-1 as the predominant cause of pulmonary hypertension related to hereditary haemorrhagic telangiectasia. J Med Genet.

[B4] Geraci MW, Moore M, Gesell T, Yeager ME, Alger L, Golpon H, Gao B, Loyd JE, Tuder RM, Voelkel NF (2001). Gene expression patterns in the lungs of patients with primary pulmonary hypertension: a gene microarray analysis. Circ Res.

[B5] Fishman AP (2000). Changing concepts of the pulmonary plexiform lesion. Physiol Res.

[B6] Diatchenko L, Lau YF, Campbell AP, Chenchik A, Moqadam F, Huang B, Lukyanov S, Lukyanov K, Gurskaya N, Sverdlov ED, Siebert PD (1996). Suppression subtractive hybridization: a method for generating differentially regulated or tissue-specific cDNA probes and libraries. Proc Natl Acad Sci U S A.

[B7] Mason NA, Springall DR, Burke M, Pollock J, Mikhail G, Yacoub MH, Polak JM (1998). High expression of endothelial nitric oxide synthase in plexiform lesions of pulmonary hypertension. J Pathol.

[B8] Thomson JR, Machado RD, Pauciulo MW, Morgan NV, Humbert M, Elliott GC, Ward K, Yacoub M, Mikhail G, Rogers P, Newman J, Wheeler L, Higenbottam T, Gibbs JS, Egan J, Crozier A, Peacock A, Allcock R, Corris P, Loyd JE, Trembath RC, Nichols WC (2000). Sporadic primary pulmonary hypertension is associated with germline mutations of the gene encoding BMPR-II, a receptor member of the TGF-beta family. J Med Genet.

[B9] Heath D, Edwards JE (1958). The pathology of hypertensive pulmonary vascular disease; a description of six grades of structural changes in the pulmonary arteries with special reference to congenital cardiac septal defects. Circulation.

[B10] Atkinson C, Stewart S, Upton PD, Machado R, Thomson JR, Trembath RC, Morrell NW (2002). Primary pulmonary hypertension is associated with reduced pulmonary vascular expression of type II bone morphogenetic protein receptor. Circulation.

[B11] Cool CD, Stewart JS, Werahera P, Miller GJ, Williams RL, Voelkel NF, Tuder RM (1999). Three-dimensional reconstruction of pulmonary arteries in plexiform pulmonary hypertension using cell-specific markers. Evidence for a dynamic and heterogeneous process of pulmonary endothelial cell growth. Am J Pathol.

[B12] Lee SD, Shroyer KR, Markham NE, Cool CD, Voelkel NF, Tuder RM (1998). Monoclonal endothelial cell proliferation is present in primary but not secondary pulmonary hypertension. J Clin Invest.

[B13] Girard JP, Springer TA (1996). Modulation of endothelial cell adhesion by hevin, an acidic protein associated with high endothelial venules. J Biol Chem.

[B14] Hambrock HO, Nitsche DP, Hansen U, Bruckner P, Paulsson M, Maurer P, Hartmann U (2003). SC1/hevin. An extracellular calcium-modulated protein that binds collagen I. J Biol Chem.

[B15] Sullivan MM, Sage EH (2004). Hevin/SC1, a matricellular glycoprotein and potential tumor-suppressor of the SPARC/BM-40/Osteonectin family. Int J Biochem Cell Biol.

[B16] Claeskens A, Ongenae N, Neefs JM, Cheyns P, Kaijen P, Cools M, Kutoh E (2000). Hevin is down-regulated in many cancers and is a negative regulator of cell growth and proliferation. Br J Cancer.

[B17] Mendis DB, Ivy GO, Brown IR (2000). Induction of SC1 mRNA encoding a brain extracellular matrix glycoprotein related to SPARC following lesioning of the adult rat forebrain. Neurochem Res.

[B18] St Croix B, Rago C, Velculescu V, Traverso G, Romans KE, Montgomery E, Lal A, Riggins GJ, Lengauer C, Vogelstein B, Kinzler KW (2000). Genes expressed in human tumor endothelium. Science.

[B19] Nareyeck G, Seidler DG, Troyer D, Rauterberg J, Kresse H, Schonherr E (2004). Differential interactions of decorin and decorin mutants with type I and type VI collagens. Eur J Biochem.

[B20] Westergren-Thorsson G, Antonsson P, Malmstrom A, Heinegard D, Oldberg A (1991). The synthesis of a family of structurally related proteoglycans in fibroblasts is differently regulated by TFG-beta. Matrix.

[B21] Border WA, Noble NA, Yamamoto T, Harper JR, Yamaguchi Y, Pierschbacher MD, Ruoslahti E (1992). Natural inhibitor of transforming growth factor-beta protects against scarring in experimental kidney disease. Nature.

[B22] Schonherr E, Sunderkotter C, Schaefer L, Thanos S, Grassel S, Oldberg A, Iozzo RV, Young MF, Kresse H (2004). Decorin deficiency leads to impaired angiogenesis in injured mouse cornea. J Vasc Res.

[B23] Nelimarkka L, Salminen H, Kuopio T, Nikkari S, Ekfors T, Laine J, Pelliniemi L, Jarvelainen H (2001). Decorin is produced by capillary endothelial cells in inflammation-associated angiogenesis. Am J Pathol.

[B24] Grant DS, Yenisey C, Rose RW, Tootell M, Santra M, Iozzo RV (2002). Decorin suppresses tumor cell-mediated angiogenesis. Oncogene.

[B25] Jarvelainen H, Vernon RB, Gooden MD, Francki A, Lara S, Johnson PY, Kinsella MG, Sage EH, Wight TN (2004). Overexpression of decorin by rat arterial smooth muscle cells enhances contraction of type I collagen in vitro. Arterioscler Thromb Vasc Biol.

[B26] Fischer JW, Steitz SA, Johnson PY, Burke A, Kolodgie F, Virmani R, Giachelli C, Wight TN (2004). Decorin promotes aortic smooth muscle cell calcification and colocalizes to calcified regions in human atherosclerotic lesions. Arterioscler Thromb Vasc Biol.

[B27] Pavloff N, Staskus PW, Kishnani NS, Hawkes SP (1992). A new inhibitor of metalloproteinases from chicken: ChIMP-3. A third member of the TIMP family. J Biol Chem.

[B28] Leco KJ, Waterhouse P, Sanchez OH, Gowing KL, Poole AR, Wakeham A, Mak TW, Khokha R (2001). Spontaneous air space enlargement in the lungs of mice lacking tissue inhibitor of metalloproteinases-3 (TIMP-3). J Clin Invest.

[B29] Gill SE, Pape MC, Khokha R, Watson AJ, Leco KJ (2003). A null mutation for tissue inhibitor of metalloproteinases-3 (Timp-3) impairs murine bronchiole branching morphogenesis. Dev Biol.

[B30] Lepetit H, Eddahibi S, Fadel E, Frisdal E, Munaut C, Noel A, Humbert M, Adnot S, D'Ortho MP, Lafuma C (2005). Smooth muscle cell matrix metalloproteinases in idiopathic pulmonary arterial hypertension. Eur Respir J.

[B31] Levchenko T, Aase K, Troyanovsky B, Bratt A, Holmgren L (2003). Loss of responsiveness to chemotactic factors by deletion of the C-terminal protein interaction site of angiomotin. J Cell Sci.

[B32] Huminiecki L, Gorn M, Suchting S, Poulsom R, Bicknell R (2002). Magic roundabout is a new member of the roundabout receptor family that is endothelial specific and expressed at sites of active angiogenesis. Genomics.

[B33] Park KW, Morrison CM, Sorensen LK, Jones CA, Rao Y, Chien CB, Wu JY, Urness LD, Li DY (2003). Robo4 is a vascular-specific receptor that inhibits endothelial migration. Dev Biol.

[B34] Seth P, Lin Y, Hanai J, Shivalingappa V, Duyao MP, Sukhatme VP (2005). Magic roundabout, a tumor endothelial marker: Expression and signaling. Biochem Biophys Res Commun.

[B35] Suchting S, Heal P, Tahtis K, Stewart LM, Bicknell R (2005). Soluble Robo4 receptor inhibits in vivo angiogenesis and endothelial cell migration. Faseb J.

[B36] Sainson RC, Aoto J, Nakatsu MN, Holderfield M, Conn E, Koller E, Hughes CC (2005). Cell-autonomous notch signaling regulates endothelial cell branching and proliferation during vascular tubulogenesis. Faseb J.

[B37] Bedell VM, Yeo SY, Park KW, Chung J, Seth P, Shivalingappa V, Zhao J, Obara T, Sukhatme VP, Drummond IA, Li DY, Ramchandran R (2005). roundabout4 is essential for angiogenesis in vivo. Proc Natl Acad Sci U S A.

[B38] Dohi Y, Ohashi M, Sugiyama M, Takase H, Sato K, Ueda R (2003). Circulating thrombomodulin levels are related to latent progression of atherosclerosis in hypertensive patients. Hypertens Res.

[B39] Rabausch K, Bretschneider E, Sarbia M, Meyer-Kirchrath J, Censarek P, Pape R, Fischer JW, Schror K, Weber AA (2005). Regulation of thrombomodulin expression in human vascular smooth muscle cells by COX-2-derived prostaglandins. Circ Res.

[B40] Barreiro O, Yanez-Mo M, Sala-Valdes M, Gutierrez-Lopez MD, Ovalle S, Higginbottom A, Monk PN, Cabanas C, Sanchez-Madrid F (2005). Endothelial tetraspanin microdomains regulate leukocyte firm adhesion during extravasation. Blood.

[B41] Tachibana I, Hemler ME (1999). Role of transmembrane 4 superfamily (TM4SF) proteins CD9 and CD81 in muscle cell fusion and myotube maintenance. J Cell Biol.

[B42] Kao YR, Shih JY, Wen WC, Ko YP, Chen BM, Chan YL, Chu YW, Yang PC, Wu CW, Roffler SR (2003). Tumor-associated antigen L6 and the invasion of human lung cancer cells. Clin Cancer Res.

[B43] Berra E, Benizri E, Ginouves A, Volmat V, Roux D, Pouyssegur J (2003). HIF prolyl-hydroxylase 2 is the key oxygen sensor setting low steady-state levels of HIF-1alpha in normoxia. Embo J.

[B44] Epstein AC, Gleadle JM, McNeill LA, Hewitson KS, O'Rourke J, Mole DR, Mukherji M, Metzen E, Wilson MI, Dhanda A, Tian YM, Masson N, Hamilton DL, Jaakkola P, Barstead R, Hodgkin J, Maxwell PH, Pugh CW, Schofield CJ, Ratcliffe PJ (2001). C. elegans EGL-9 and mammalian homologs define a family of dioxygenases that regulate HIF by prolyl hydroxylation. Cell.

[B45] Knowles HJ, Tian YM, Mole DR, Harris AL (2004). Novel mechanism of action for hydralazine: induction of hypoxia-inducible factor-1alpha, vascular endothelial growth factor, and angiogenesis by inhibition of prolyl hydroxylases. Circ Res.

[B46] Rossetti S, Hoogeveen AT, Sacchi N (2004). The MTG proteins: chromatin repression players with a passion for networking. Genomics.

[B47] Gergely F, Kidd D, Jeffers K, Wakefield JG, Raff JW (2000). D-TACC: a novel centrosomal protein required for normal spindle function in the early Drosophila embryo. Embo J.

[B48] Still IH, Hamilton M, Vince P, Wolfman A, Cowell JK (1999). Cloning of TACC1, an embryonically expressed, potentially transforming coiled coil containing gene, from the 8p11 breast cancer amplicon. Oncogene.

[B49] Miller JW, Urbinati CR, Teng-Umnuay P, Stenberg MG, Byrne BJ, Thornton CA, Swanson MS (2000). Recruitment of human muscleblind proteins to (CUG)(n) expansions associated with myotonic dystrophy. Embo J.

[B50] Ho TH, Charlet BN, Poulos MG, Singh G, Swanson MS, Cooper TA (2004). Muscleblind proteins regulate alternative splicing. Embo J.

[B51] Nakamura T, Sanokawa R, Sasaki YF, Ayusawa D, Oishi M, Mori N (1995). Cyclin I: a new cyclin encoded by a gene isolated from human brain. Exp Cell Res.

[B52] Dickinson LA, Edgar AJ, Ehley J, Gottesfeld JM (2002). Cyclin L is an RS domain protein involved in pre-mRNA splicing. J Biol Chem.

[B53] Berke JD, Sgambato V, Zhu PP, Lavoie B, Vincent M, Krause M, Hyman SE (2001). Dopamine and glutamate induce distinct striatal splice forms of Ania-6, an RNA polymerase II-associated cyclin. Neuron.

[B54] Johnson ME, Viggiano L, Bailey JA, Abdul-Rauf M, Goodwin G, Rocchi M, Eichler EE (2001). Positive selection of a gene family during the emergence of humans and African apes. Nature.

[B55] Oksvold MP, Huitfeldt HS, Langdon WY (2004). Identification of 14-3-3zeta as an EGF receptor interacting protein. FEBS Lett.

[B56] Mangin P, David T, Lavaud V, Cranmer SL, Pikovski I, Jackson SP, Berndt MC, Cazenave JP, Gachet C, Lanza F (2004). Identification of a novel 14-3-3zeta binding site within the cytoplasmic tail of platelet glycoprotein Ibalpha. Blood.

[B57] Mishra SK, Hawryluk MJ, Brett TJ, Keyel PA, Dupin AL, Jha A, Heuser JE, Fremont DH, Traub LM (2004). Dual engagement regulation of protein interactions with the AP-2 adaptor alpha appendage. J Biol Chem.

[B58] Chow VT, Quek HH (1996). HEP-COP, a novel human gene whose product is highly homologous to the alpha-subunit of the yeast coatomer protein complex. Gene.

[B59] Feurle GE (1998). Xenin--a review. Peptides.

[B60] Gupta A, Hruska KA, Martin KJ (1994). Neurotensin binding to human embryonic lung fibroblasts. J Recept Res.

[B61] Edgar AJ, Polak JM (2000). Human homologues of yeast vacuolar protein sorting 29 and 35. Biochem Biophys Res Commun.

[B62] Li S, Armstrong CM, Bertin N, Ge H, Milstein S, Boxem M, Vidalain PO, Han JD, Chesneau A, Hao T, Goldberg DS, Li N, Martinez M, Rual JF, Lamesch P, Xu L, Tewari M, Wong SL, Zhang LV, Berriz GF, Jacotot L, Vaglio P, Reboul J, Hirozane-Kishikawa T, Li Q, Gabel HW, Elewa A, Baumgartner B, Rose DJ, Yu H, Bosak S, Sequerra R, Fraser A, Mango SE, Saxton WM, Strome S, Van Den Heuvel S, Piano F, Vandenhaute J, Sardet C, Gerstein M, Doucette-Stamm L, Gunsalus KC, Harper JW, Cusick ME, Roth FP, Hill DE, Vidal M (2004). A map of the interactome network of the metazoan C. elegans. Science.

[B63] Favre CJ, Mancuso M, Maas K, McLean JW, Baluk P, McDonald DM (2003). Expression of genes involved in vascular development and angiogenesis in endothelial cells of adult lung. Am J Physiol Heart Circ Physiol.

[B64] Jaleel M, McBride A, Lizcano JM, Deak M, Toth R, Morrice NA, Alessi DR (2005). Identification of the sucrose non-fermenting related kinase SNRK, as a novel LKB1 substrate. FEBS Lett.

[B65] Ylikorkala A, Rossi DJ, Korsisaari N, Luukko K, Alitalo K, Henkemeyer M, Makela TP (2001). Vascular abnormalities and deregulation of VEGF in Lkb1-deficient mice. Science.

[B66] Perry DK, Hannun YA (1998). The role of ceramide in cell signaling. Biochim Biophys Acta.

[B67] Andrews MT, Squire TL, Bowen CM, Rollins MB (1998). Low-temperature carbon utilization is regulated by novel gene activity in the heart of a hibernating mammal. Proc Natl Acad Sci U S A.

[B68] Holness MJ, Kraus A, Harris RA, Sugden MC (2000). Targeted upregulation of pyruvate dehydrogenase kinase (PDK)-4 in slow-twitch skeletal muscle underlies the stable modification of the regulatory characteristics of PDK induced by high-fat feeding. Diabetes.

[B69] Sugden MC, Kraus A, Harris RA, Holness MJ (2000). Fibre-type specific modification of the activity and regulation of skeletal muscle pyruvate dehydrogenase kinase (PDK) by prolonged starvation and refeeding is associated with targeted regulation of PDK isoenzyme 4 expression. Biochem J.

[B70] Wu P, Sato J, Zhao Y, Jaskiewicz J, Popov KM, Harris RA (1998). Starvation and diabetes increase the amount of pyruvate dehydrogenase kinase isoenzyme 4 in rat heart. Biochem J.

[B71] Hind M, Corcoran J, Maden M (2002). Alveolar proliferation, retinoid synthesizing enzymes, and endogenous retinoids in the postnatal mouse lung. Different roles for Aldh-1 and Raldh-2. Am J Respir Cell Mol Biol.

[B72] Hough RB, Piatigorsky J (2004). Preferential transcription of rabbit Aldh1a1 in the cornea: implication of hypoxia-related pathways. Mol Cell Biol.

[B73] Whetstine JR, Yueh MF, McCarver DG, Williams DE, Park CS, Kang JH, Cha YN, Dolphin CT, Shephard EA, Phillips IR, Hines RN (2000). Ethnic differences in human flavin-containing monooxygenase 2 (FMO2) polymorphisms: detection of expressed protein in African-Americans. Toxicol Appl Pharmacol.

[B74] Dolphin CT, Beckett DJ, Janmohamed A, Cullingford TE, Smith RL, Shephard EA, Phillips IR (1998). The flavin-containing monooxygenase 2 gene (FMO2) of humans, but not of other primates, encodes a truncated, nonfunctional protein. J Biol Chem.

[B75] Eddahibi S, Raffestin B, Hamon M, Adnot S (2002). Is the serotonin transporter involved in the pathogenesis of pulmonary hypertension?. J Lab Clin Med.

[B76] Eddahibi S, Humbert M, Fadel E, Raffestin B, Darmon M, Capron F, Simonneau G, Dartevelle P, Hamon M, Adnot S (2001). Serotonin transporter overexpression is responsible for pulmonary artery smooth muscle hyperplasia in primary pulmonary hypertension. J Clin Invest.

[B77] Bull TM, Coldren CD, Moore M, Sotto-Santiago SM, Pham DV, Nana-Sinkam SP, Voelkel NF, Geraci MW (2004). Gene microarray analysis of peripheral blood cells in pulmonary arterial hypertension. Am J Respir Crit Care Med.

[B78] Dorfmuller P, Perros F, Balabanian K, Humbert M (2003). Inflammation in pulmonary arterial hypertension. Eur Respir J.

[B79] Golpon HA, Coldren CD, Zamora MR, Cosgrove GP, Moore MD, Tuder RM, Geraci MW, Voelkel NF (2004). Emphysema lung tissue gene expression profiling. Am J Respir Cell Mol Biol.

[B80] Tuder RM, Chacon M, Alger L, Wang J, Taraseviciene-Stewart L, Kasahara Y, Cool CD, Bishop AE, Geraci M, Semenza GL, Yacoub M, Polak JM, Voelkel NF (2001). Expression of angiogenesis-related molecules in plexiform lesions in severe pulmonary hypertension: evidence for a process of disordered angiogenesis. J Pathol.

